# Ginsenoside Re Regulates Oxidative Stress through the PI3K/Akt/Nrf2 Signaling Pathway in Mice with Scopolamine-Induced Memory Impairments

**DOI:** 10.3390/cimb46100677

**Published:** 2024-10-13

**Authors:** Xin Li, Kai Zheng, Hao Chen, Wei Li

**Affiliations:** College of Chinese Medicinal Materials, Jilin Agricultural University, Changchun 130118, China

**Keywords:** ginsenoside Re, scopolamine, PI3K/Akt/Nrf2 signaling pathway, oxidative stress, apoptosis

## Abstract

While Ginsenoside Re has been shown to protect the central nervous system, reports of its effects on memory in the model of scopolamine-induced memory impairment are rare. The aim of this study was to investigate the effects of Ginsenoside Re on scopolamine (SCOP)-induced memory damage and the mechanism of action. Male ICR mice were treated with SCOP (3 mg/kg) for 7 days and with or without Ginsenoside Re for 14 days. As evidenced by behavioral studies (escape latency and cross platform position), brain tissue morphology, and oxidative stress indicators after Ginsenoside Re treatment, the memory damage caused by SCOP was significantly ameliorated. Further mechanism research indicated that Ginsenoside Re inhibited cell apoptosis by regulating the PI3K/Akt/Nrf2 pathway, thereby exerting a cognitive impairment improvement effect. This research suggests that Ginsenoside Re could protect against SCOP-induced memory defects possibly through inhibiting oxidative stress and cell apoptosis.

## 1. Introduction

Alzheimer’s disease is a neurodegenerative disorder characterized by the progressive degeneration of neurons in the brain, particularly in regions associated with memory, language, and cognitive functions. This neuronal damage results in the manifestation of initial symptoms such as memory loss, language difficulties, and cognitive impairment. Alzheimer’s disease is characterized by its progressive nature, resulting in a deterioration of cognitive abilities over time. The rate of progression and specific cognitive impairments experienced can vary among individuals. As the disease advances, neuronal damage increases and various regions of the brain become affected [1,2]. The aging population of China is experiencing an increase in the prevalence of Alzheimer’s disease and other neurodegenerative diseases. The incidence and mortality rates of Alzheimer’s disease have risen dramatically in recent years, ranking fifth among the country’s leading causes of mortality for both urban and rural residents. This disease imposes a heavy economic burden on individuals, families, and society [3]. 

One of the primary contributors to Alzheimer’s disease is the presence of oxidative imbalance in the central nervous system, which disrupts metabolic processes. Reactive oxygen species (ROS) are generated as a byproduct of various cellular processes, particularly those involving mitochondria. When oxidative imbalance occurs and ROS are not adequately regulated, cellular damage ensues, resulting in a range of disruptions to metabolic processes [4]. Abnormal cellular metabolism in turn affects the production and accumulation of amyloid beta (Aβ) and hyperphosphorylated Tau protein, which independently exacerbate mitochondrial dysfunction and ROS production, leading to the destruction of organelles and structures, thereby contributing to a vicious cycle [5,6]. Ultimately, cells may malfunction or undergo apoptosis as the redox balance shifts towards oxidative conditions [7]. Scopolamine is one of the main drugs used in experimental models of AD that induce cognitive deficits in animals [8]. Many changes can be caused by scopolamine, including abnormalities in the cholinergic system, oxidative stress imbalance, mitochondrial dysfunction, and cell death [9].

It has long been known that the active ingredients in natural medicinal plants or their active ingredients can delay aging and treat neurodegenerative disease due to their diverse effects, non-toxicity, and potential benefits [10,11]. As a kind of traditional Chinese medicine, Panax ginseng C. A. Meyer has been widely used to treat various diseases for thousands of years [12,13,14]. Research indicates that ginseng has both pharmacological properties and almost no side effects; therefore, it is regarded as a functional food and supplement around the world [15]. Various active ingredients have been isolated from ginseng in modern research, such as ginsenosides, polysaccharides, monosaccharides, vitamins, amino acids, organic acids, etc. [16,17]. Ginsenoside Re is one of the most abundant ginsenosides in ginseng and has good antioxidant properties [18]. Ginsenoside Re exhibits pharmacological effects in both in vitro and in vivo in models of Alzheimer’s disease. Research has shown that Ginsenoside Re can alleviate the peroxidation reaction of SH-SY5Y cells, reduce Aβ levels in N2a/APP695 cells, and improve cognitive function in mice [19,20,21]. These studies suggest that Ginsenoside Re may be an ideal drug for treating neurodegenerative diseases such as Alzheimer’s disease. However, the neuroprotective mechanism of Ginsenoside Re is still unclear and needs to be further explored. 

The purpose of this study was to explore the protective effect of Ginsenoside Re by establishing a SCOP-induced mouse AD model to further reveal the possible molecular mechanism.

## 2. Materials

Ginsenoside Re was provided from the College of Chinese Medicinal Materials, Jilin Agricultural University, with a purity of >98%, formulated into the required concentration with 0.5% sodium carboxymethyl cellulose; scopolamine (NO.SO20230308-0014C642651343D10) was purchased from Aladdin, Shanghai, China. 

The reagent kits for superoxide dismutase (SOD) (NO. 20230829), malondialdehyde (MDA) (NO. 20230829), and nitric oxide (NO) (NO. 20230829) were purchased from Nanjing Jiancheng Bioengineering Institute (Nanjing, China). Primary antibodies against caspase 9 (NO. WL01551), cleaved-caspase 9 (NO. 10380-1-AP), caspase 3 (NO. A11888), cleaved-caspase 3 (NO. YM3431), Bax (NO. Y70445), cytochrome c (NO. 66264-1-IG), Bad (NO. 67830-1-IG), Bak (NO. 21552-1-AP), Bcl-2 (NO. YM3041), Bcl-xL (NO. 26967-1-AP), p-PI3K (NO. 67071-1-IG), PI3K (NO. 20584-1-AP), AKT (NO. 10176-2-AP), p-AKT (NO. 66444-1-IG), GSK-3β (NO. 22104-1-AP), p-GSK-3β (NO. 14850-1-AP), Nrf2 (NO. YT3189), HO-1 (NO. 10701-1-AP), and Keap1 (NO.10503-2-AP) were purchased from Proteintech (Wuhan, China) and Immunoway (Beijing, China). β-actin (NO. AB179467) was purchased from Abcam (Shanghai, China). Goat anti-rabbit IgG secondary antibody HRP (NO. SAB L3032) and HRP-conjugated goat anti-rat IgG (NO. AS028) were purchased from Signalway Antibody (Greenbelt, MD, USA) and ABclonal (Wuhan, China). The hematoxylin and eosin (H&E) dying kit and the DAPI staining kit were obtained from Beyotime Co, Ltd. (Shanghai, China). TUNEL apoptosis detection kits were provided by Roche Applied Science (Shanghai, China). 

### 2.1. Animals

Eight-week-old male ICR mice, weighing 22–25 g, were purchased Changchun YISI Experimental Animal Holding with a Certificate of Quality No. SCXK (JI) 2016-0003 (Changchun, China). The animals were housed in a sterile environment with controlled temperature (23.0 ± 2.0 °C) and humidity (60 ± 10%) in a 12 h light/dark pattern. Sufficient water and food were provided. All experimental procedures were approved by the Ethical Committee for Laboratory Animals of Jilin Agricultural University (Permit Number: 20–008). We made every effort to minimize suffering of the animals.

### 2.2. Experimental Design 

The mice were allowed to acclimatize and received the same diet before and during the experiment. After one week of adaptive feeding, mice were randomly assigned into four groups (*n* =10 each): the normal group, SCOP group (SCOP dose, 3 mg/kg), low-dose Re group (SCOP + Re 10 mg/kg), and the high-dose Re group (SCOP + Re 20 mg/kg). Generally, the recommended modeling dose for mice is 3 mg/kg per day intraperitoneal injection (i.p.) for 7 days, which has been shown to result in a significant reduction in their learning and memory abilities [22,23]. The whole experiment lasted for 14 days, and the animal experiment included two ways of administration: gavage and injection. The details are as follows: Mice in the normal group were given daily intragastric administration of 0.5% sodium carboxymethyl cellulose solution (0.5% CMC-Na) in the same amount as each group for 14 days, and intraperitoneal injection of the same amount of normal saline was given in 8–14 days. Mice in the SCOP group were given 0.5% CMC-Na daily for 14 days and intraperitoneal injection of 3 mg/kg/d of SCOP on days 8–14. Mice in the low-dose Re group were given an intragastric dose of 10 mg/kg of Re (dissolved in 0.5%CMC-Na) daily, and intraperitoneal injection of 3 mg/kg/d of SCOP on days 8–14. Mice in the high-dose Re group were given an intragastric dose of 20 mg/kg of Re (dissolved in 0.5%CMC-Na) daily, and were given an intraperitoneal dose of 3 mg/kg/d of SCOP on days 8–14. The Morris water maze test was conducted 30 min after SCOP injection. It included an orientation navigation experiment and a space exploration experiment. All experimental animals were euthanized after the last MWM. Blood and brain tissue samples were immediately collected. The mice brains were sagittally cut in half, and the brains were dissected and fixed in 10% neutral buffered formalin for 24 h at 4 °C. After fixation, some of the samples were dehydrated and embedded in OCT and kept at −80 °C until further analysis.

### 2.3. Morris Water Maze (MWM)

MWM analysis was used to evaluate brain functions of mice’s spatial learning and memory abilities. It was filled with opaque water (22 °C) in the Morris water maze apparatus (21–23 °C). There were four quadrants in the Morris water maze apparatus, one of which was equipped with a circular escape platform (6 cm in diameter), and water was added to the pool until the water level was 1 cm above the platform. The training started from the 10th day. The mice were placed in the pool during training, where they could swim freely to find the escape platform. The time that the mice took to find the platform was recorded. Whenever mice could not find the platform within 120 s, the mice were guided gently to the platform and kept there for 30 s. The mice were trained four times a day during the training period. After 4 consecutive days of training, we removed the platform on the 14th day and put the mice into the pool to swim. The software WMT-100(Smart 3.0, Panlab, Spain) of Taimeng Technology Co., Ltd. was used to keep records of relevant data (Chengdu, China).

### 2.4. Detection of Biochemical Parameters in Mice

Brain tissues from each group were homogenized on ice, and serum samples were separated by refrigerated centrifuge at 3000× *g*. The supernatant was collected and examined immediately using the assay kits as directed by the manufacturer’s instructions (Nanjing Jiancheng Bioengineering Institute, Nanjing, China).

### 2.5. Histopathology Examination

Brain tissues were soaked in 10% formalin solution for more than 24 h. Then, the samples were dehydrated with ethanol gradient and embedded in paraffin. The paraffin-embedded brain tissues were sliced into sections of 5 μm and stained with H&E staining kits and photographed using optical microscopy (Leica TCS SP8, Solms, Germany). 

### 2.6. TUNEL and DAPI Staining

Detection of apoptotic cells was performed using TUNEL and DAPI staining as directed by the manufacturer’s protocol. The slices were dewaxed and then fixed with protease K (20 mg/mL) for 30 min. In order to block endogenous peroxidase, the brain tissues were incubated in methanol with 3% H_2_O_2_ for 20 min. Equilibration buffer and terminal deoxynucleotidyl transferase were used to treat the sections. Then, the slices were treated with anti-digoxin-peroxidase conjugate. The peroxidase activity in each brain slice was stained with DAB and counter-stained with hematoxylin. DAPI staining was performed after washing three times with phosphate-buffered saline (0.01 M, pH 7.4). Then, images were taken of the stained nuclei under UV excitation using a fluorescence microscope. The stained nuclei were excited by UV and photographed under by fluorescence microscopy. Images were captured by a Leica microscope (Leica TCS SP8, Solms, Germany). 

### 2.7. Western Blotting Analysis

The brain tissue from each group was placed in ice-cold lysis buffer. Equal amounts of protein from each sample were placed into an SDS-PAGE gel to separate the proteins, and then the separated proteins were transferred to a PVDF membrane. The target band was blocked with 5% skim milk for 2.5 h and incubated with the specific primary antibody for 2 h. The primary antibodies used in this study were as follows: Caspase 9 (1:1000), cleaved-caspase 9 (1:1000), caspase 3 (1:1000), cleaved-caspase 3 (1:1000), Bax (1:1000), cytochrome c (1:1000), Bad (1:1000), Bak (1:1000), Bcl-2 (1:1000), Bcl-xL (1:1000), p-PI3K (1:1000), PI3K (1:1000), AKT (1:1000), p-AKT (1:1000), GSK-3β (1:1000), p-GSK-3β (1:1000), Nrf2 (1:1000), HO-1 (1:1000), and Keap1 (1:1000). Then, the membrane was washed 3 times with TBS·T (TBS + 0.4% Tween-20) and incubated with the secondary antibody for 2 h. Eventually, the Tanon 5200 Multi System (Tanon Biotechnology Co., Ltd., Shanghai, China) was used to detect and analyze chemiluminescent signals. The intensity of each protein band was analyzed using Quantity One software(4.6.8) (Bio-Rad Laboratories, Hercules, CA, USA).

### 2.8. Statistical Analysis

An analysis of the data was performed using SPSS 21.0 Statistics, presenting the mean ± standard deviation (SPSS, Inc., Chicago, IL, USA). Statistical differences across groups were assessed using one-way analysis of variance (ANOVA) followed by Scheffe’s test. Statistical significance was defined as *p* values less than 0.05 or 0.01. Statistical tests were performed using software of GraphPad Prism 9.3.1 software (GraphPad Software, Inc., San Diego, CA, USA)

## 3. Results

### 3.1. Efficacy of Ginsenoside Re on the Learning and Memory Damage Caused by SCOP in AD Mice

**Figure 1:**
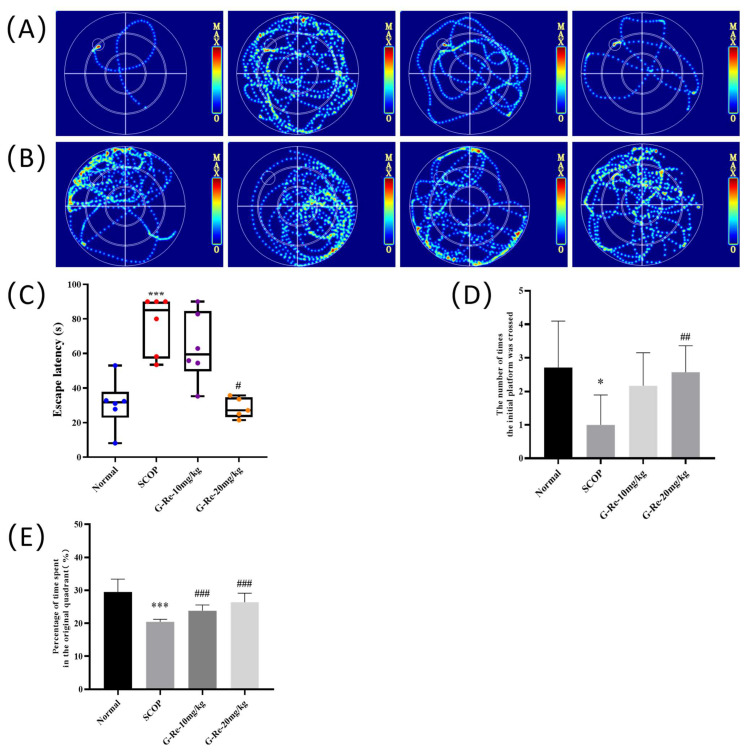
Effects of Ginsenoside Re treatment on SCOP-induced memory impairment in mice. (**A**) Training trial swim trajectory for escape latency at the fifth day. (**B**) Swimming trajectory after the probe trial was conducted without the platform. (**C**) The latency to reach the hidden platform. (**D**) The number of times the initial platform was crossed during probe test. (**E**) Percentage of time spent in the original quadrant. Data are presented as mean ± S.D. (n = 6). * *p* < 0.05, *** *p* < 0.001 compared with the normal group; # *p* < 0.05, ## *p* < 0.01, ### *p* < 0.001 compared with SCOP group.

Figure 1A,B below illustrate that mice in the normal group could quickly find the hidden platform, swim over the target quadrant, and make multiple crossings of the hidden platform’s position. Compared with the normal group, the escape latency of SCOP group mice was significantly prolonged (*p* < 0.01) (Figure 1C), the target quadrant residence time was significantly reduced (*p* < 0.05) (Figure 1E), and the number of cross platform positions was significantly reduced (*p* < 0.05) (Figure 1D). These results indicated that intraperitoneal injection of SCOP could lead to learning and memory damage in mice. Compared with the SCOP group, after treatment with Ginsenoside Re, the escape latency of SCOP group mice was significantly shortened (*p* < 0.05), the target quadrant residence time was significantly increased (*p* < 0.05), and the number of cross platform positions was significantly increased (*p* < 0.05) (Figure 1C–E). The results indicated that Ginsenoside Re reversed SCOP-induced spatial and memory damage in a significant way. 

### 3.2. Effects of Ginsenoside Re on SOD Activity, MDA Content, and NO Content in Brain Tissues of Mice

**Figure 2:**
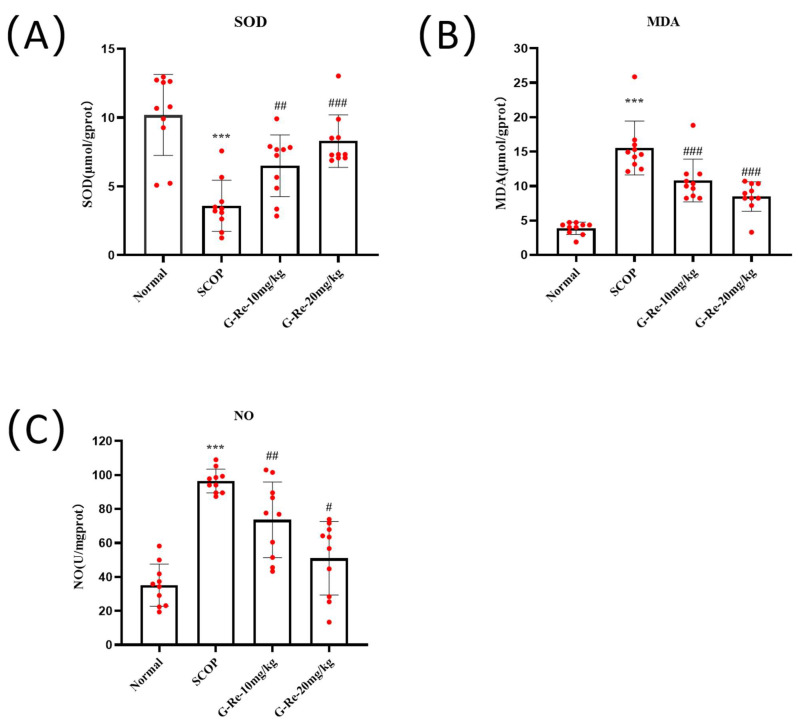
Efficacy of Ginsenoside Re in reducing oxidative stress in mice with SCOP-induced memory damage. (**A**–**C**) Levels of SOD, MDA, and NO in brain tissues. Data are presented as mean ± S.D. (n = 10). *** *p* < 0.001 compared with the normal group; # *p* < 0.05, ## *p* < 0.01, ### *p* < 0.001 compared with SCOP group.

As shown in Figure 2, compared with the normal group, there was a significant decrease in SOD activity following SCOP, accompanied by increases in MDA and NO contents. As compared to the SCOP group, administration of Ginsenoside Re significantly increased SOD activity after and also reduced MDA and NO contents (*p* < 0.05). These results fully demonstrated that Ginsenoside Re could effectively recover antioxidant capacity and prevent oxidative damage to brain tissues through modulation of oxidative stress. 

### 3.3. Effects of Ginsenoside Re on Hippocampal Neuron Morphology

**Figure 3:**
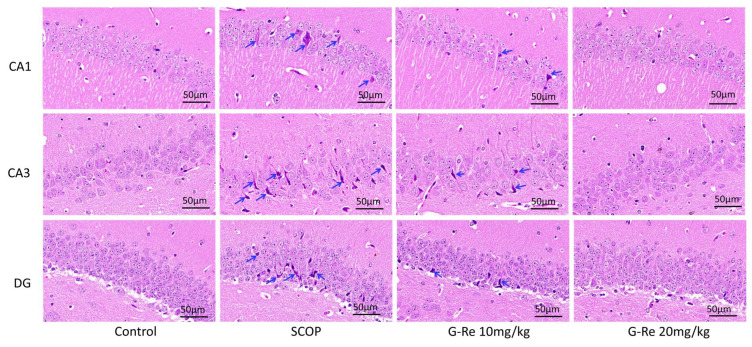
Changes in the histopathology of mice with SCOP-induced memory damage following the administration of Ginsenoside Re. H&E was applied to the brain sections after Morris water maze. Shown are some representative images of H&E-stained brain sections from the test 40×). Data are presented as mean ± S.D. (n = 3).

Damage to brain function inevitably leads to varying degrees of pathological damage. Therefore, we further confirmed the protective effect of Ginsenoside Re on mice brain memory function damage through H&E staining analysis. As shown in Figure 3, we found greater numbers of hippocampal neuron cells had regular morphology, clear layers, and neat arrangement in the normal group. Compared with the normal group, the number of hippocampal neuron cells of mice in the SCOP group was significantly reduced. The arrangement of these hippocampal neuron cells was loose. These hippocampal neuron cells were not neatly arranged, and there were some discontinuous parts among the cell layer and gaps among the cells. Furthermore, some cells exhibited generally disrupted morphology, stained nuclei, and pyknosis occurred. Compared with the SCOP group, after administering Ginsenoside Re, the number of hippocampal neurons increased, and the arrangement of hippocampal neurons restored. Cell nuclei pyknosis improved and nucleoli were obvious. 

### 3.4. Effects of Ginsenoside Re on Neuronal Apoptosis

**Figure 4:**
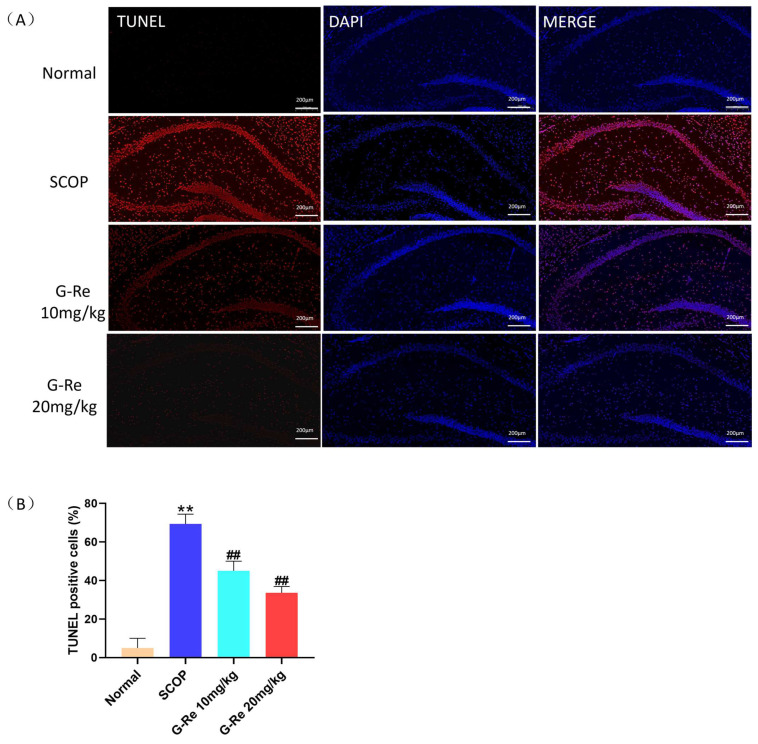
Changes in brain tissues in mice with SCOP-induced memory damage after consuming Ginsenoside Re. (**A**) Sections of the brain were stained with TUNEL and DAPI after the behavioral test. (**B**) The presence of TUNEL positive cells were measured by the image analyzer. Data are presented as mean ± S.D. (n = 3). ** *p* < 0.01 compared with the normal group; ## *p* < 0.01 compared with SCOP group.

In order to further confirm that Ginsenoside Re treatment can alleviate SCOP induced neuronal apoptosis, we used TUNEL and DAPI staining to observe the degree of neuronal apoptosis combined with TUNEL staining to quantitatively detect the level of apoptosis. As shown in Figure 4A,B, compared with the normal group, the fluorescence intensity in the hippocampus of the SCOP group mice increased and the degree of neuronal apoptosis significantly increased. This fully demonstrates that SCOP can induce apoptosis in neuronal cells. Compared with the SCOP group, the number of fluorescent-stained neurons in the hippocampus of mice was significantly reduced, and the fluorescence intensity was detected to decrease after treatment with Ginsenoside Re. According to DAPI staining, the nuclei of the normal group were intact and mostly round and light blue in shape. However, most cells had undergone apoptosis and aggregated into bright blue fluorescence in the SCOP group. This indicated severe neuronal apoptosis after SCOP treatment. Compared with the SCOP group, neuronal cell apoptosis was alleviated and neuronal damage was effectively improved after treatment with Ginsenoside Re. 

### 3.5. Effects of Ginsenoside Re on the Expression Levels of Apoptosis-Related Proteins

**Figure 5:**
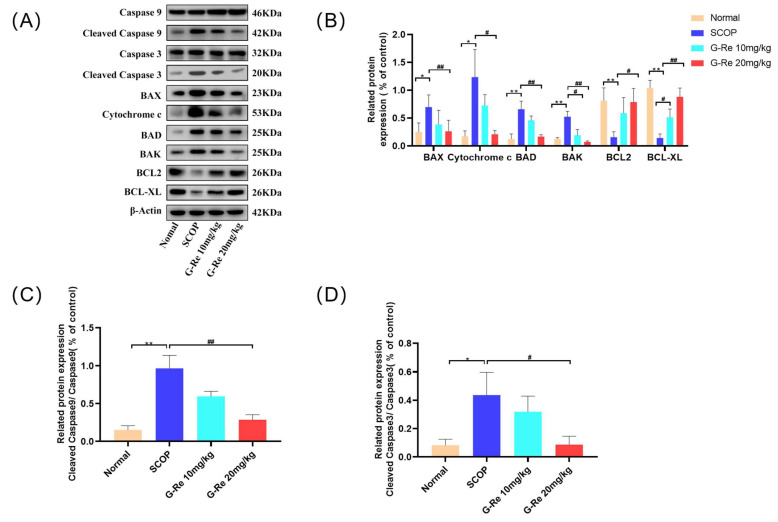
Effects of Ginsenoside Re on apoptotic proteins. (**A**) Densitometry was used to measure the relative expression and level of key apoptotic participants. (**B**–**D**) Western blot analysis for Bad, Bak, Bax, Bcl-2, Bcl-xL, cytochrome c, cleaved-caspase 3 and cleaved-caspase 9. Data are presented as mean ± S.D. (n = 3). * *p* < 0.05, ** *p* < 0.01 compared with the normal group; # *p* < 0.05, ## *p* < 0.01 compared with SCOP group.

To further assess the degree of cell apoptosis, Western blotting analysis was performed on apoptosis-related proteins. As shown in Figure 5, compared with the normal group, the expressions of pro-apoptotic proteins Bax, Bad, cytochrome c, and Bak in brain tissue significantly increased, while the expressions of antiapoptotic Bcl-xL and Bcl-2 significantly decreased after SCOP treatment. On the contrary, compared with the SCOP group, Ginsenoside Re could significantly upregulate the expression of Bcl-xL and Bcl-2 antiapoptotic proteins while reducing the levels of Bax, Bad, cytochrome c, and Bak pro-apoptotic proteins. These results fully demonstrated that Ginsenoside Re could inhibit the expressions of SCOP-induced apoptotic proteins. 

### 3.6. Effects of Ginsenoside Re on the Expression of PI3K/AKT Signaling Pathway-Related Factors

**Figure 6:**
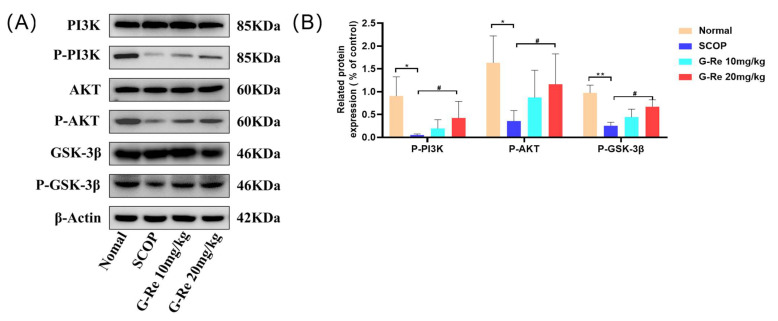
Effects of Ginsenoside Re on PI3K/AKT signaling pathway. (**A**) Densitometry was used to measure the relative expressions of key PI3K/AKT signaling participants. (**B**) Western blot analysis for P-AKT, P-GSK3β, and P-PI3K. Data are presented as mean ± S.D. (n = 3). * *p* < 0.05, ** *p* < 0.01 compared with the normal group; # *p* < 0.05 compared with SCOP group.

In order to further explore the molecular mechanism of Ginsenoside Re against SCOP-induced brain damage, we detected the relative expressions of key PI3K/AKT signaling participants via Western blotting. As shown in Figure 6, compared with the normal group, the abundance of p-PI3K, p-AKT, and p-GSK3β decreased after treatment with SCOP. As compared to the SCOP group, we found that Ginsenoside Re treatment could increase the expressions of p-PI3K, p-AKT, and p-GSK3β in brain tissues. These results fully demonstrated that Ginsenoside Re could activate the PI3K/AKT signaling pathway. 

### 3.7. Effects of Ginsenoside Re on the Expression of Nrf2 Signaling Pathway-Related Factors

**Figure 7:**
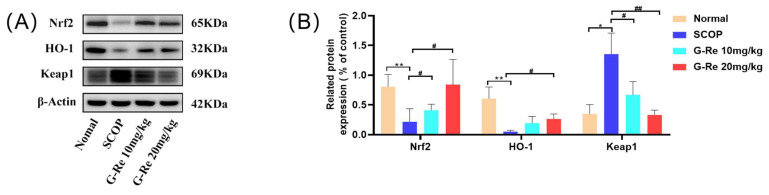
Effects of Ginsenoside Re on Nrf2 signaling pathway. (**A**) Densitometry was used to measure the relative expressions of Nrf2, HO-1, and Keap1. (**B**) Western blot analysis for Nrf2, HO-1, and Keap1. Data are presented as mean ± S.D. (n = 3). * *p* < 0.05, ** *p* < 0.01 compared with the normal group; # *p* < 0.05, ## *p* < 0.01 compared with SCOP group.

To further explore the oxidative stress effects of Ginsenoside Re on the mechanism of cell apoptosis, we evaluated the expression levels of Nrf2, Keap1, and HO-1 using Western blotting analysis. As shown in Figure 7, compared with the normal group, transduction of the Nrf2/Keap1 signaling pathway was inhibited after SCOP treatment, and the expressions of Nrf2 protein and HO-1 protein were significantly reduced, while the expression of Keap1 protein was significantly increased. Compared with the SCOP group, the expressions of Nrf2 protein and HO-1 protein were significantly increased, while the expression of Keap1 protein was significantly reduced. These results indicated that Ginsenoside Re could reduce oxidative stress induced by SCOP to protect brain tissue.

## 4. Discussion

Research on the pathogenesis of AD has found that its pathogenic factors are very complex, which poses certain difficulties for the development of AD treatment drugs, but also provides opportunities for studying the role of natural drugs in AD [24,25]. Panax ginseng C. A. Meyer, which is well known for its active ingredients (especially ginsenosides), has shown good neuroprotective properties through various mechanisms and has no side effects [26]. Pharmacological effects of Ginsenoside Re are currently being studied extensively, with broad potential and possible applications. 

We aimed to determine the neuroprotective properties and mechanisms of Ginsenoside Re in this study. Our model was based on SCOP-induced AD mice to elucidate the efficacy of Ginsenoside Re on memory impairment. Apoptosis and oxidative stress were enhanced in model mice injected with SCOP, which resembled clinical symptoms of Alzheimer’s disease [27]. As indicated by the literature, SCOP can be used in animal studies to investigate memory and learning impairments [28]. Spatial memory was tested by the Morris water maze to evaluate rodent learning and memory [29]. In this study, behavioral analysis was combined with memory testing to explore the potential effects of Re in improving learning and memory impairment in mice induced by SCOP. The results suggest that Ginsenoside Re intervention showed significant effects on many defects associated with SCOP, including dysregulation of the PI3K/AKT signaling pathway and Nrf2/Keap1 signaling pathway, poor performance in the Morris water maze, and high levels of oxidative stress.

The Morris water maze was considered as important indicators to evaluate learning and memory [30]. Behavioral trials showed that animals exposed to SCOP showed increased escape latency, decreased staying time in the quadrant of the platform, and reduced cross platform position frequency, indicating impaired memory in mice. Ginsenoside Re treatment significantly reversed the above symptoms, indicating that Ginsenoside Re rescued memory impairment caused by SCOP.

Brain function damage will inevitably lead to different degrees of pathological damage in the brain [31]. According to pathological results, the structure of neurons in the hippocampal region of the model group was looser, and the density of neurons decreased; the neurons were smaller, and the cytoplasm was darker. Furthermore, nuclei appeared to be a certain degree of staining in deep blue or were even ruptured. The above pathological changes can be alleviated to a certain degree following Ginsenoside Re treatment.

Alzheimer’s disease is complex and not fully understood, but growing evidence suggests that loss of neurons in the cortex and hippocampus accounts for most of the cognitive decline [32]. Under pathological conditions, apoptosis is believed to be the primary cause of AD-related manifestations, resulting in abnormal neuronal loss, which is closely related to the degree of AD and may precede progression of the disease [33]. The apoptotic process involves a number of proteins, including members of the Bcl-2 family, such as Bax, Bad, Bcl-2, Bcl-xL, and caspase, and proceeds through a series of events, ultimately resulting in the disintegration of cells [34]. The Bcl-2 protein family plays a crucial role in cell health, mainly acting on mitochondria and inhibiting and promoting apoptosis in cells at the same time [35]. Apoptosis of neural cells during central nervous system development is triggered by Bak and Bax proteins, which release cytochrome C and encode caspase 3 [36]; Bcl-2 and Bcl-xL belong to the Bcl-2 family of antiapoptotic proteins that prevent apoptosis during brain development [37]. Functions of cytochrome C divided into survival and stress signaling functions [38]. When the process goes wrong, the result could be mitochondrial dysfunction leading to various health problems, including neurodegeneration, ischemia-reperfusion injury, and cancer [39]. Caspases are a family of evolutionary conserved cysteine-dependent endonucleases; due to their role in tissue differentiation, regeneration, and neural development, as well as the relationship with inducing apoptosis in cells, caspases have received considerable attention [40]. Apoptosis occurs when cytochrome C is released from mitochondria, activating caspase 9 and downstream executioners caspase 3 [41]. PI3K/AKT is an upstream regulatory factor of GSK-3β, and GSK-3β is a downstream kinase of the PI3K/AKT signaling pathway [42]. When PI3K is phosphorylated to p-Pi3K, p-Akt and p-GSK-3β are also activated at serine 473 and serine 9, respectively, which is consistent with previous reports [43]. GSK-3 β is abundant in the brain and is associated with many neurodegenerative and neurological diseases such as Alzheimer’s disease, Parkinson’s disease, etc. Specifically, inhibiting GSK-3 β in neuronal cells has been shown to protect neural progenitor cells from genetic toxicity and other stress-induced cell apoptosis [44]. Phosphorylation of Ser9 sites can ultimately lead to inactivation of GSK-3β [45]. P-GSK-3β inhibits mitochondrial translocation of the pro-apoptotic molecule Bax and degradation of the antiapoptotic molecule Bcl-2 to prevent cell apoptosis [46]. Previous studies have shown that in postmortem AD brain samples, the levels of p-PI3K, p-Akt, and p-GSK-3β were decreased [47]. The results we observed match description in the literature; Ginsenoside Re treatment reduced neuronal apoptosis, improved neuronal damage, and increased the levels of p-PI3K, p-AKT, and p-GSK3β in brain tissues. Ginsenoside Re treatment can maintain the homeostasis of mouse brain tissue and inhibit excessive apoptosis of neuronal cells. 

The central nervous system (CNS) is highly susceptible to oxidative damage due to increased oxygen consumption [48]. However, CNS cells are particularly susceptible to oxidative stress due to their abundance of readily oxidizable substances, low antioxidant levels, and various internal mechanisms that generate ROS, resulting in redox imbalance shifts to oxidation, leading to mitochondrial dysfunction, apoptosis, and cell damage, ultimately triggering neurodegenerative processes [49]. The brain consumes the highest amount of oxygen, which is a cause of oxidative damage resulting in increased lipid peroxidation and decline in other antioxidant markers [50]. Oxidative damage leads to an increase in lipid peroxides (such as MDA and NO), a decrease in antioxidant marker SOD activity, and causes DNA damage and mitochondrial oxidative damage by targeting cell membrane permeability [51,52,53]. Scopolamine has been shown to affect oxidative stress levels in the brain and lead to learning and memory deficits in animals. Scopolamine alters the levels of SOD and MDA in mice, which is associated with oxidative stress [54]. As with previous studies, scopolamine has been shown to affect oxidative stress levels in the brain by altering the antioxidant defense system, altering the levels of enzymes related to oxidative stress such as SOD, MDA, and NO in mice, and leading to learning and memory deficits in animals [55]. Nrf2/Keap1 signaling is a key mechanism for protecting cells against oxidative stress damage, and the body activates this pathway in response to oxidative stress to initiate antioxidant mechanisms [56]. Nrf2 is a kind of redox-sensitive transcription factor that can trigger cellular antioxidant responses and protect cells from intrinsic or extrinsic oxidative stress [57]. Keap1 is inhibited physiologically when Nrf2 binds to it in the cytoplasm [58]. When stimulated by external oxidative stress molecules or nucleophilic substances leading to an increase in ROS levels, Nrf2 is decoupled from Keap1 [59]. Nrf2 is then activated and enters the nucleus to bind with the antioxidant response element (ARE). Subsequently, it promotes the genetic levels of downriver antioxidative enzymes (HO-1 and SOD, etc.) to prevent various sources of oxidative stress and reduce tissue damage [60]. SCOP can induce neuronal damage and cell apoptosis, and more evidence suggests that in the endogenous antioxidant defense system Nrf2-mediated antioxidant signaling pathways are crucial for the prevention of cell injury and apoptosis resulting from oxidative stress [61,62,63]. In the endogenous apoptotic signaling pathway, cytochrome c is a key regulatory factor that activates caspase-dependent cell apoptosis and triggers the activation of caspase 3, ultimately leading to cell death [64]. The release of cytochrome C is mainly influenced by the pro-apoptotic genes of the Bcl-2 family proteins [65]. The Bcl-2 family proteins not only include pro-apoptotic factors, but also anti-apoptotic factors, which maintain cellular homeostasis through the regulation of the Bcl-2 family [66]. The results we observed match description in the literature, SCOP-reduced activity of oxidative stress marker SOD, and increased levels of MDA and NO in the brain of SCOP mice, leading to oxidative damage; however, Ginsenoside Re reversed these changes. In our study, the results indicate that the regulation of p-PI3K activity leads to an increase in the phosphorylation activity of downstream p-Akt and p-GSK-3β. At the same time, we found that Ginsenoside Re inhibited the expressions of pro-apoptotic proteins such as Bax and caspase family proteins and promoted the expression of anti-apoptotic proteins Bcl-2 and Bcl-xL. Additionally, Ginsenoside Re treatment promoted mobile expression of Nrf2, which promoted downstream expression of HO-1 while reducing Keap1 expression in brain tissues. Ginsenoside Re can regulate the endogenous pathway-related factors of brain tissue cells in mice, maintain brain tissue homeostasis in mice, and inhibit excessive neuronal apoptosis. 

PI3K/AKT is an upstream regulator of GSK-3β, and GSK-3β is a downstream kinase. The abnormal activation of GSK-3β is closely related to aging and neurodegenerative diseases. Nrf2 is a downstream target of GSK-3β and a key molecule in signaling between PI3K/Akt/GSK-3β and antioxidant stress [67]. Thus, we believe that Ginsenoside Re plays an important role in AD-related diseases by activating the PI3K/Akt/Nrf2 cascade reaction. 

In summary, Ginsenoside Re has a protective effect on SCOP-induced AD mice. These effects may be mainly attributed to Re’s inhibition of oxidative stress and cell apoptosis by regulating the PI3K/AKT/GSK3β pathway. These findings enrich the research on the pharmacological function of ginsenosides for Alzheimer’s disease, and also provide a basis for the development of Alzheimer’s disease drugs.

## Data Availability

The research data used to support the findings of this study are included in the article.
